# Aneurism of the Arch of the Aorta

**Published:** 1858-07

**Authors:** Benj. H. Lemon


ART. VII.—*Aneurism of the Arch of the Aorta. Probable Death by "Embolic Apoplexy," from Detachment of Fibrinous Coagula.* Case of Dr. GOULD. Dissection three days after death, before Dr. NOTT, Coroner; Present, Professor HAMILTON, and FLINT, Drs. WILCOX, ORTON, and Medical Students. Dissection and Report by BENJ. H. LEMON, M. D.

Alfred Taylor, a negro marine cook, aged about forty years, first observed a tumor at the upper part of the chest, to the right of the sternum, about two years ago. At the time of his death, November, 1857, it protruded externally to about the size of a man's heart, neither thrill nor murmurs could be felt or heard in it. For several months, he has been unable to bear the recumbent posture, in consequence of the difficulty of respiration induced by it, he was constantly restless, often walking the room.

The dyspnœa gradually increased, and in November, after more distress than usual, he rose from his bed to walk the room, he had crossed the floor but a few times, when he fell and shortly died.

On dissection, three days after death, we found a "*fusiform partially sacculated aneurism of the arch of the Aorta*," about as large as a medium sized cocoa-nut, the dilatation commencing one and a half inches above the bases of the semilunar valves, continuing progressively and involving the whole of the arch of the Aorta, including the innominata nearly up to the roots of the subclavian and carotid arteries, its walls being so expanded, as to be incorporated into the general parietes of the aneurismal sac, the right and left subclavian and carotid arteries were given off from the superior aspect of the tumor and were normal. The sac was entire.

The lateral half of the lower end of the superior portion of the sternum, together with the cartilage, and extremity of the second rib was absorbed. The extremity of the third rib was denuded of periosteum and with the cartilage, also partially absorbed.

The integument covering the first, second, and third intercostal spaces was very thin, that of the second, apparently, very soon to have given way, and alone with the remainder of the extremity and cartilage of the third rib, formed the external wall of the sac, the proper coats of the vessel having given way in front for the space of several square inches, and formed adhesions above and below to the thoracic parietes, at which points there were projecting ridges; all parts of the sac, except anteriorly, were lined by a red membrane, the proper internal serous coat of the vessel; anteriorly, a deposit of ragged fibrinous coagula was quite firmly attached; indeed, with this substance and clotted blood, the sac was nearly filled, containing, probably, more than a pint.

The valves were healthy, as was also the heart throughout, though hypertrophied. The specimen is preserved in the collection of "Prof. Flint," College Museum. The investigation was pursued no further.

"As to the immediate cause of death, Prof. Flint was of the opinion that fragments of the coagula had become detached, been carried into the circulation, and obstructed the vessels of the neck and head, thereby causing, what has been lately termed, '*Embolic Apoplexy*.'

Of this pathological condition, or the liability to this accident of those having aneurism of the arch of the Aorta innommate cr carotid arteries, even the latest works on pathological anatomy seem to make no mention.

Rokitansky says, "Aneurism very commonly terminates fatally; amongst special causes we may place diffused inflammation terminating in gangrene, dropsy of the cavities of the body, hyperœmia and acute œdema, more especially of the lungs, cachexia, and general marasmus." For a similar case see the American Journal of the Medical Sciences, for January 1858, by Prof. Esmarch, in which the danger of much manipulation of aneurismal tumors is dwelt on, as being liable to cause detachment of coagula.

